# CRISPR/Cas9-mediated editing of *eukaryotic elongation factor 1B gamma* (*eEF1Bγ*) reduces *Tobacco etch virus* accumulation in *Nicotiana benthamiana*

**DOI:** 10.1007/s00299-025-03440-x

**Published:** 2025-02-22

**Authors:** Bomi Kang, Jelli Venkatesh, Joung-Ho Lee, Jung-Min Kim, Jin-kyung Kwon, Byoung-Cheorl Kang

**Affiliations:** 1 Interdisciplinary Program in Agricultural Biotechnology, College of Agriculture and Life Sciences, Seoul National University, Seoul, 08826 Republic of Korea; 2 Department of Agriculture, Forestry and Bioresources, Research Institute of Agriculture and Life Sciences, Plant Genomics Breeding Institute, College of Agriculture and Life Sciences, Seoul National University, Seoul, 08826 Republic of Korea; 3 FarmyirehSe Co., Ltd., Seoul, 08826 Republic of Korea

**Keywords:** CRISPR/Cas9, Host factor, Eukaryotic elongation factor 1B γ subunit (eEF1Bγ), *Tobacco etch virus* (TEV), *Nicotiana benthamiana*

## Abstract

**Key message:**

*Tobacco etch virus* accumulation declined in *Nicotiana benthamiana eEF1Bγ* gene-edited lines, suggesting that eEF1Bγ may be a host factor for this virus.

**Abstract:**

Viruses use host factors to replicate and move from cell to cell. Therefore, the editing of genes encoding viral host factors that are not essential for plant survival enables the rapid development of plants with durable virus resistance. Eukaryotic initiation factors, such as eIF4E and eIF4G, function as host factors for viral infection, and loss-of-function mutations of these factors lead to virus resistance. Broadening the spectrum of host factor targets would help expand resources for engineering virus resistance. In this study, we tested whether editing the eukaryotic translation elongation factor gene *eEF1Bγ* would produce virus-resistant plants. Accordingly, we targeted the four *eEF1Bγ* genes in *Nicotiana benthamiana* for editing using virus-induced gene editing (VIGE) with *Tobacco rattle virus* (TRV). Although we attempted to obtain plants edited for all four *eEF1Bγ* homologs, we failed to identify such plants. Instead, we obtained plants with three of the four homologs knocked out, harboring 1-bp insertion/deletions resulting in premature stop codons. These *eEF1Bγ*-edited plants did not exhibit resistance to *Potato virus X* (PVX), *Tobacco mosaic virus* (TMV), or *Tomato bushy stunt virus* (TBSV) but showed reduced accumulation of *Tobacco etch virus* (TEV) compared to wild-type plants. These findings demonstrate the feasibility of conferring resistance in plants through gene editing of *eEF1Bγ*, underscoring the importance of exploring diverse host factor targets for comprehensive virus resistance.

**Supplementary Information:**

The online version contains supplementary material available at 10.1007/s00299-025-03440-x.

## Introduction

Diseases caused by plant viruses pose a significant threat to global agriculture, causing substantial economic losses (Mumford et al. 2016). Various breeding methods have been used to develop virus-resistant cultivars, with one common approach being the introgression of resistance (*R*) genes from wild relatives into elite cultivars (Kumar 1999; Kang et al. 2005a). However, conventional breeding methods are time consuming and labor intensive, as they often require extensive backcrossing to introgress desired traits into elite backgrounds. Moreover, the emergence of new viral strains that overcome existing resistance adds to the challenge of deploying genetic resistance, especially when the available germplasm lacks traits that confer resistance to the new strains. Plant RNA viruses exploit host factors for their replication and movement (Garcia‐Ruiz 2019). Gene editing of viral host factors can confer recessive resistance, which is considered to be more durable than resistance conferred by dominant* R* genes (Kang et al. 2005a; Hashimoto et al. 2016).

Eukaryotic translation initiation factors (eIFs) are essential in assembling the translation initiation complex and recognizing start codons, while eukaryotic translation elongation factor (eEFs) facilitate chain elongation by adding amino acids. eIF4E and eIF4G in the eIF4F complex are critical for translation initiation, with eIF4E binding to the mRNA 5' cap and eIF4G serving as a scaffold, linking eIF4A for ribosome recruitment (Browning and Bailey-Serres 2015). eIF2 delivers the initiator tRNA to the ribosome. For RNA viruses such as members of the Potyviridae, host translation factors such as eIFs play important roles in viral replication. Indeed, genetic studies of recessive resistance genes and studies using gene editing have shown that the loss-of-function of eIF4E (Bastet et al. 2017, 2019; Gomez et al. 2019; Yoon et al. 2020; Lucioli et al. 2022; Noureen et al. 2022; Pechar et al. 2022; Kan et al. 2023) and eIF4G can confer virus resistance (Macovei et al. 2018; Wang et al. 2021a). Nevertheless, expanding the repertoire of host factor targets offers the potential for breeding virus-resistant plant varieties that can combat a broader spectrum of viruses.

Like eIFs, eEFs have also been linked to virus resistance. Eukaryotic translation elongation factor 1 (eEF1) is composed of two major components: eEF1A, which delivers aminoacyl-tRNAs to the ribosome; and eEF1B, which acts as a nucleotide exchange factor (Sasikumar et al. 2012). In plants, eEF1B consists of the structural protein eEF1Bγ, along with two nucleotide exchange subunits: eEF1Bα and eEF1Bβ (Le Sourd et al. 2006). Although there are currently no findings of natural resistance resources based on eEF1B mutations, studies using virus-induced gene silencing (VIGS) have implicated eEF1B in RNA virus replication. Suppressing *eEF1B* using VIGS in *Nicotiana benthamiana* and pepper (*Capsicum annuum*) resulted in reduced accumulation and spread of *Tobacco mosaic virus* (TMV), with eEF1B shown to interact directly with the methyltransferase domain of TMV RNA-dependent RNA polymerase (RdRp) together with eEF1A, suggesting that it may act as a host factor for TMV (Hwang et al. 2013). In addition, suppressing *eEF1Bβ* or *eEF1Bγ* expression in *N. benthamiana* led to a significantly decreased accumulation of *Potato virus* X (PVX) (Hwang et al. 2015). In addition, deletion of *eEF1Bγ* resulted in reduced replication of *Tomato bushy stunt virus* (TBSV) in a yeast host (*Saccharomyces cerevisiae*), and knockdown of *eEF1Bγ* in *N. benthamiana* led to a decrease in TBSV and TMV RNA replication (Sasvari et al. 2011). Given its potential role as a host factor for multiple RNA viruses, eEF1Bγ represents a promising target for gene editing to explore its ability to confer virus resistance.

In this study, we employed gene editing to mutate *eEF1Bγ* genes in *N. benthamiana* in an effort to obtain virus-resistant plants. Gene editing via Clustered regularly interspaced short palindromic repeats/CRISPR-associated protein 9 (CRISPR/Cas9) systems has emerged as a promising strategy to rapidly create resistance alleles (Chandrasekaran et al. 2016). However, the difficulty in delivering reagents and the need to regenerate transformed plants have hampered efforts to use this technology. Virus-induced gene editing (VIGE) uses viral vectors to deliver single-guide RNAs (sgRNAs) into *Cas9*-overexpressing plants, allowing gene-edited plants to be produced without the need for tissue culture (Ali et al. 2015; Oh et al. 2021). Furthermore, VIGE using mobile RNA sequences, such as the *FLOWERING LOCUS T* (*FT*) transcript or isoleucine transfer RNA (tRNA-Ile) attached to the sgRNA allows the sgRNA to move between plant cells, thus enhancing the editing efficiency of this system (Ellison et al. 2020; Lei et al. 2021; Chen et al. 2022; Nagalakshmi et al. 2022).

Here, we used VIGE to produce *eEF1Bγ*-edited lines in *N*. *benthamiana* and tested the resistance of these lines to four different viruses: PVX, TMV, TBSV, and *Tobacco etch virus* (TEV). We determined that the four *eEF1Bγ* homologs in *N*. *benthamiana* likely play an essential role and that eEF1Bγ is a host factor for TEV. Moreover, our observations support the efficacy of gene editing in conferring resistance to viral infections.

## Materials and methods

### Expression analysis of *eEF1Bγ* in *N. benthamiana*

To confirm the expression of the *eEF1Bγ* homologs, three SRA files (SRR11747765, SRR11747766, and SRR11747767) corresponding to *N. benthamiana* leaf samples (Wang et al. 2021b) were downloaded from NCBI Sequence Read Archive (SRA) database. To normalize for sequencing depth and gene length, the Fragments Per Kilobase per Million mapped fragments (FPKM) values were calculated for four *eEF1Bγ* transcripts (Niben101Scf10540g00002.1, Niben101Scf01552g05010.1, Niben101Scf01552g05013.1, and Niben101Scf03350g00002.1). The FPKM values provide a measure of transcript abundance in each sample, allowing for the confirmation of whether the *eEF1Bγ* transcripts are expressed in the leaf tissues.

### TRV-sgRNA design and vector construction for gene editing

To verify successful editing via restriction enzyme digestion, the sgRNA target sequence was designed to include the *Hpy*166II restriction enzyme recognition site within the target region. A BLAST search was performed on the Sol Genomics Network, USA, to ensure that the designed sgRNA target site would simultaneously target all *eEF1Bγ* homologs in *N. benthamiana* and to confirm the absence of off-target effects. Subsequently, the sgRNA targeting the *eEF1Bγ* homologs was amplified using the pTRV2-NbPDS-sgRNA construct (Lee et al. 2024) as a template. The amplified *eEF1Bγ*-sgRNA PCR product was then purified using a LaboPass PCR Clean-up kit (Cosmo Genetech, Seoul, Republic of Korea) following the manufacturer’s instructions and digested with *Mfe*I and *Xma*I restriction enzymes to create compatible overhangs. The digested sgRNA fragment was ligated into the corresponding *Mfe*I and *Xma*I restriction sites of the pTRV2 vector (Table S1) using T4 DNA ligase (NEB, Beverly, MA, USA). The ligated product was then transformed into competent *Escherichia coli* DH5α cells (BIOFACT, Daejeon, Republic of Korea). Positive colonies were selected on a LB agar plate containing 50 µg/mL kanamycin and verified by colony PCR.

### Agrobacterium-mediated TRV inoculation

For Agrobacterium (*Agrobacterium tumefaciens*)-mediated infiltration of TRV into *N. benthamiana* leaves, the pTRV2-*eEF1Bγ*-sgRNA vector was transformed into Agrobacterium strain GV3101 by electroporation. Following electroporation, the transformed Agrobacterium cells were recovered in LB broth at 28 °C for 3 h with shaking at 200 rpm, and then plated on LB agar containing 50 µg/mL kanamycin and 50 µg/mL rifampicin to select positive colonies. Positive Agrobacterium colonies were inoculated in LB medium and grown overnight at 28 °C with shaking at 200 rpm. On the following day, the cultures were collected by centrifugation and resuspended in infiltration buffer (10 mM MgCl_2_, 10 mM MES, 200 µM acetosyringone, pH 5.6) with OD_600_ adjusted to 0.6. Following incubation at room temperature for 3 h with shaking, the Agrobacterium cell suspension harboring the pTRV2 was mixed with Agrobacterium cell suspensions carrying the pTRV1 vector or the P19 silencing suppressor at a 1:1:1 ratio (v/v/v). The mixed suspensions were infiltrated into the abaxial side of the leaves of 4-week-old *Cas9* transgenic *N. benthamiana* plants using a 1 mL needleless syringe. The *Cas9*-overexpressing transgenic *N. benthamiana* plants were previously developed for VIGE (Lee et al. 2024). All infiltrated plants were grown in a growth chamber at 25 °C under a 16-h light/8-h dark photoperiod and 60% relative humidity.

### Genomic DNA extraction from *N. benthamiana*

Genomic DNA (gDNA) was extracted from the *N. benthamiana* samples using the cetyltrimethylammonium bromide (CTAB) method as previously described (Porebski et al. 1997). The concentration and purity of the extracted gDNA were determined using a Nanodrop spectrophotometer (Nanodrop Technology, Inc., Wilmington, Delaware, USA), and the gDNA was diluted to a final concentration of 50 ng/μL for use in subsequent PCR reactions.

### Detection of mutations

The *eEF1Bγ* fragment was amplified from the gDNA samples by PCR using primers flanking the target sites of each *eEF1Bγ* homolog (Table S1). The PCR products were purified using either a LaboPass PCR Clean-up kit (Cosmo Genetech, Seoul, Republic of Korea) or AMPure XP (BECKMAN COULTER, Brea, CA, USA) following the respective manufacturer’s instructions. The purified *eEF1Bγ* PCR products were digested overnight at 37 °C with *Hpy*166II (NEB, Beverly, MA, USA), which specifically recognizes the *eEF1Bγ* target region. The digested PCR products were analyzed on a 1.5% (w/v) agarose gel, and the ratio of uncut to cut bands was calculated using ImageJ software (NIH and LOCI) to quantify the editing efficiency at each target site. The PCR products were also cloned using an All-in-One PCR Cloning Kit (BIOFACT, Daejeon, Republic of Korea) following the manufacturer’s instructions and sent for Sanger sequencing (Macrogen, Daejeon, Republic of Korea). The resulting DNA sequences were aligned and compared with the WT sequences using the Lasergene’s SeqMan program (DNASTAR, Madison, WI) to identify the mutated sites.

To verify off-target effects, potential off-target sites for the *eEF1Bγ* target site were predicted using the CRISPOR tool (http://crispor.gi.ucsc.edu/). Among these, seven predicted target sites with ≤ 3-nt mismatches and a cutting frequency determination (CFD) score ≥ 0.2 were sequenced individually for mutation screening (Table S3).

### Regeneration of TRV-inoculated *N. benthamiana*

Systemically infected leaves were collected at 2–3 weeks after TRV inoculation and surface sterilized by first immersing them in 70% (v/v) ethanol for 30 s and 10% (v/v) commercial bleach (with a drop of Tween 20) for 20 min. The leaves were then washed four times with sterile water. Sterilized leaves were cut into 1-cm longitudinal sections, and the leaf fragments were placed onto non-selective regeneration medium (4.4 g/L Murashige and Skoog [MS] medium salts including vitamins, 30 g/L sucrose, 1 mg/L benzyl aminopurine [BAP], 0.1 mg/L indole-3-acetic acid [IAA], 8 mg/L micro-agar, pH 5.8). Regenerated shoots were excised and transferred to rooting medium (4.4 g/L MS medium salts including vitamins, 30 g/L sucrose, 8 mg/L micro-agar, pH 5.8). After 2–3 weeks of root induction, when the regenerated plantlets reached a height of 5 cm, whole plantlets were transferred to potting soil.

### Characterization of *eEF1Bγ* homolog expression in edited lines

To assess the expression patterns of the *eEF1Bγ* homologs in the mutant plants, reverse-transcription quantitative PCR (RT-qPCR) was performed on leaves collected from 4-week-old *eEF1Bγ*-edited *N. benthamiana* plants. Total RNA was extracted using a MG Total RNA Extraction kit (MGmed, Seoul, Republic of Korea) following the manufacturer’s protocol. The quality and integrity of the extracted RNA were assessed using a Nanodrop spectrophotometer and agarose gel electrophoresis. For first-strand cDNA synthesis, 1 μg of total RNA was reverse-transcribed into cDNA using AccuPower RT PreMix (Bioneer, Daejeon, Republic of Korea). The resulting cDNA was diluted 1:4 with nuclease-free water for use in the qPCR reactions. qPCR was conducted with primers specific for each *eEF1Bγ* homolog (Table S1) using a 2X Real-Time PCR Master Mix (Biofact, Daejeon, Republic of Korea) according to the manufacturer’s instructions. Amplification was performed using a QuantStudio 3 Real-Time PCR Instrument (Thermo Fisher Scientific, Waltham, MA, USA) with the following thermal cycling conditions: an initial denaturation at 95 °C for 15 min; followed by 40 cycles of 95 °C for 10 s, 58 °C for 15 s, and 72 °C for 15 s. The relative expression levels of each homolog were normalized against *ACTIN* as a reference gene and quantified using the ΔCt method.

### Virus inoculation and evaluation of virus resistance

Frozen inocula of *Potato virus X* (PVX), *Tobacco mosaic virus* (TMV), *Tomato bushy stunt virus* (TBSV), and *Tobacco etch virus* (TEV-HAT isolate) stored at − 80 °C were used to inoculate 4-week-old *N. benthamiana* plants. The frozen inocula were ground in 0.1 M potassium phosphate buffer (pH 7.0), mixed with 400-grit carborundum, and gently rubbed onto the two lower leaves of each *N. benthamiana* plants. After an inoculation period of 10–20 min, the infected leaves were washed with distilled water. The plants were then placed in a growth chamber at 25 °C with a 16-h light/8-h dark photoperiod. To inoculate the *eEF1Bγ*-edited plants, freshly infected *N. benthamiana* leaves showing visible symptoms were collected from previously inoculated WT plants, and inocula were prepared as described above. Both inoculated and systemic leaves from each plant were harvested at 5 days post-inoculation (DPI). Virus accumulation in the leaf tissues was quantified using an enzyme-linked immunosorbent assay (ELISA) according to the manufacturer’s instructions (Agdia, Elkhart, IN, USA). The absorbance of the samples at 405 nm was measured using a MULTISKAN FC microplate photometer (Thermo Fisher Scientific, Waltham, MA, USA). For the quantitative ELISA of TEV, substrate extracts from WT *N. benthamiana* plants were diluted to 1, 1/2, 1/4, and 1/8 concentrations. The absorbance values of these dilutions were used to generate a standard curve (*R*^2^ > 0.99). TEV accumulation in each *eEF1Bγ*-edited plant was then calculated based on this standard curve. Statistical significance of virus accumulation levels relative to WT plants was determined using a two-tailed *t* test in Microsoft Excel.

## Results

### Identification of *eEF1Bγ* genes in the *N. benthamiana* genome

We hypothesized that eEF1Bγ may act as a host factor and that the mutation of *eEF1Bγ* may result in virus resistance. Before generating an *eEF1Bγ* mutant, we looked for a functional *eEF1Bγ* gene expressed in *N. benthamiana*. Based on two eEF1Bγ sequences (encoded by At1g09640 and At1g57720) from Arabidopsis (*Arabidopsis thaliana*), we obtained *eEF1Bγ* sequences from *N. benthamiana*, tomato (*Solanum lycopersicum*), and pepper (*Capsicum annuum*) through a BLASTP search of the Sol Genomics Network database (SGN, http://solgenomics.net/). We identified two homologs (Solyc06g011280.3.1 and Solyc11g028100.2.1) in tomato, which is in agreement with a previous study (Hwang et al. 2015); we identified the single copy *eEF1Bγ* gene CA11g17920 in pepper. We detected four possible eEF1Bγ homologs in *N. benthamiana* (encoded by Niben101Scf10540g00002.1, Niben101Scf01552g05010.1, Niben101Scf01552g05013.1, and Niben101Scf03350g00002.1) using BLASTP.

Phylogenetic analysis based on the eEF1Bγ amino acid sequences of all four plant species (*N. benthamiana*, Arabidopsis, tomato, and pepper) grouped the sequences into different clusters based on species (Fig. S1). Protein domain analysis indicated that each eEF1Bγ harbors two typical glutathione domains and an eEF1Bγ C-terminal domain (Fig. S2). Furthermore, the eEF1Bγ proteins from the four species share 76–98% similarity (Table S2). Within *N. benthamiana*, the alignment of each eEF1Bγ sequence revealed > 90% similarity (Table S2 and Fig. S3).

We analyzed published transcriptome data from the Sequence Read Archive (SRA) at NCBI to test whether these four *eEF1Bγ* homologs are indeed expressed genes. We mapped the SRA files SRR11747765, SRR11747766, and SRR11747767, corresponding to three replicate leaf samples, to the *N. benthamiana* reference genome to quantify expression levels (Fig. S4). The fragments per kilobase of transcript per million mapped reads (FPKM) values of each *eEF1Bγ* homolog ranged from 38.2 to 58.4, confirming that all four homologs are expressed. These results reveal that the four *NbeEF1Bγ* genes are expressed and are, therefore, suitable for gene editing.

### Generation of *eEF1Bγ*-edited plants

**Fig. 1:**
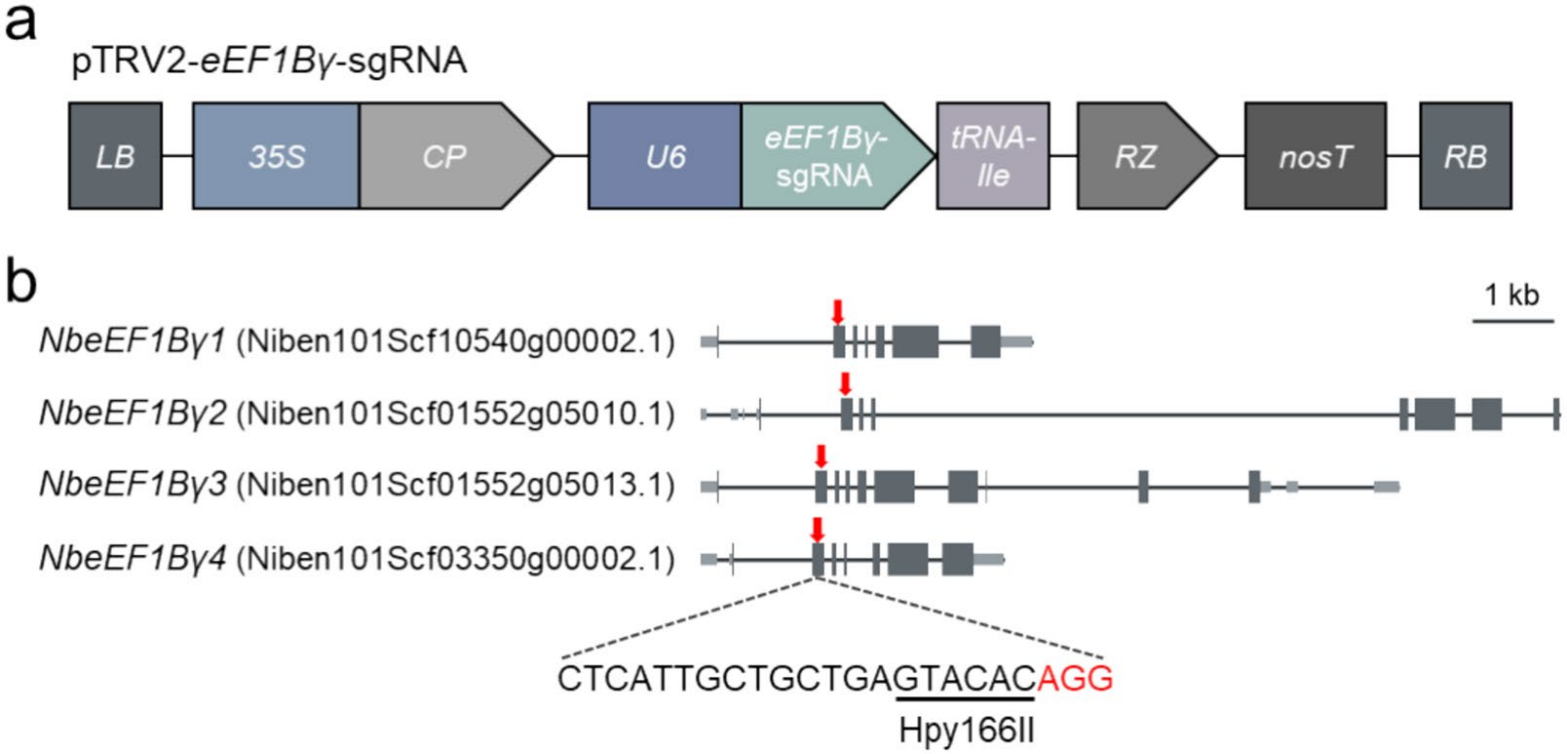
The pTRV-*eEF1Bγ*-sgRNA construct and the *eEF1Bγ* target sites. **a** Diagram of the pTRV-sgRNA construct targeting *eEF1Bγ* homologs in *N. benthamiana*. *LB* left border, *35S* cauliflower mosaic virus 35S promoter, *CP* coat protein, *U6* *AtU6-26* promoter, *RZ* terminating ribozyme, *nosT* *nopaline synthase* terminator, *RB* right border. **b** Positions of CRISPR/Cas9 target sites in the four *eEF1Bγ* homologs of *N. benthamiana*. Light-grey boxes represent the 3' and 5' UTRs, while dark-grey boxes indicate exons. Red arrows indicate the sgRNA target site in each *eEF1Bγ* homolog. The restriction site for *Hpy*166II, the enzyme used for mutation detection, is underlined in the sgRNA target site. Red font indicates the protospacer adjacent motif (PAM)

**Fig. 2:**
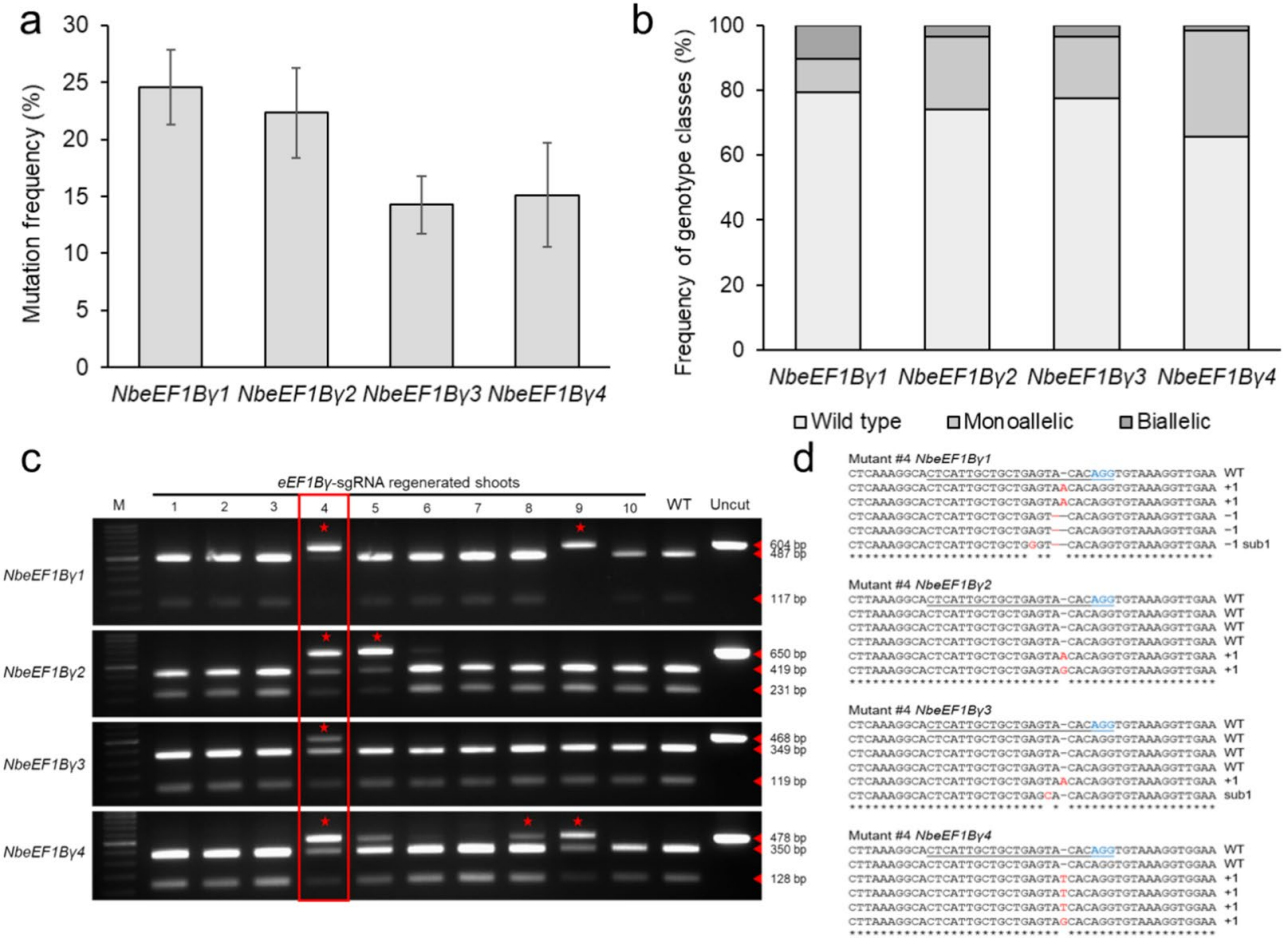
TRV-mediated gene editing of *eEF1Bγ* in *N. benthamiana*. **a** Mutation frequency in genomic DNA extracted from the systemic leaves of plants inoculated with pTRV-*eEF1Bγ*-sgRNA at 21 days post-inoculation (DPI). Data are presented as means ± standard deviation (SD). *n* = 9 plants per gene. **b** Percentage of regenerated shoots carrying mutations from pTRV-*eEF1Bγ*-sgRNA-inoculated plants. Fifty-eight regenerated shoots were examined to detect mutations. **c** Mutation detection in regenerated putative *eEF1Bγ*-edited shoots by restriction enzyme digestion. The target region of each *eEF1Bγ* homolog was amplified by PCR and digested with *Hpy*166II*.* Asterisks indicate uncut bands, indicative of mutations in the *eEF1Bγ* target sequence. All homologs were mutated in mutant #4, as indicated by the red box. **d** Multiple sequence alignment of each *eEF1Bγ* homolog across the target sites in regenerated mutant #4. Each target amplicon was cloned and subjected to Sanger sequencing. sub 1, 1-bp substitution. Red letters indicate Indels or substitution mutations. Black underlines indicate the *eEF1Bγ* target sequence. Blue letters indicate the PAM

To generate *eEF1Bγ*-edited plants, we used the VIGE method (Ali et al. 2015), in which a TRV-based vector expressing a specific sgRNA is inoculated into *Cas9*-overexpressing plants. We selected one sgRNA capable of simultaneously targeting all four homologs and constructed the corresponding pTRV-*eEF1Bγ*-sgRNA vector targeting the second exon of each homolog (Fig. 1). We attached a tRNA isoleucine sequence to the 3' end of the *eEF1Bγ*-sgRNA to facilitate cell-to-cell mobility and enhance systemic editing through the mobile sgRNA (Fig. 1a). To facilitate the detection of mutations, we chose an sgRNA target site that included a restriction site for the enzyme *Hpy*166II; this site may be destroyed in the case of successful editing (Fig. 1b). We infiltrated the pTRV-*eEF1Bγ*-sgRNA construct into previously developed *Cas9* transgenic *N. benthamiana* plants via Agrobacterium (*Agrobacterium tumefaciens*)-mediated infiltration (Lee et al. 2024). After 3 weeks, we harvested randomly selected upper leaves from the pTRV-*eEF1Bγ*-sgRNA-inoculated plants, extracted genomic DNA, and subjected it to mutation detection by genotyping PCR, restriction digestion, and Sanger sequencing. We identified mutations in each *eEF1Bγ* homolog, with mutation frequencies of 24.6% (*NbeEF1Bγ1*, Niben101Scf10540g00002.1), 22.3% (*NbeEF1Bγ2*, Niben101Scf01552g05010.1), 14.3% (*NbeEF1Bγ3*, Niben101Scf01552g05013.1), and 15.1% (*NbeEF1Bγ4*, Niben101Scf03350g00002.1) (Fig. 2a).

To generate *eEF1Bγ*-edited plants, we performed tissue culture using the systemic leaves from pTRV-*eEF1Bγ*-sgRNA-inoculated plants. Of the 58 regenerated shoots obtained, 12 (20.7%), 15 (25.9%), 13 (22.4%), and 20 (34.5%) shoots harbored mutations in *NbeEF1Bγ1*, *NbeEF1Bγ2*, *NbeEF1Bγ3*, or *NbeEF1Bγ4*, respectively (Fig. 2b). In addition, 1.7–10.3% of the shoots carried biallelic mutations in at least one homolog. We selected mutant #4, in which all homologs were edited, for generational progression to obtain biallelic mutants of all homologs (Fig. 2c). We confirmed the mutant sequences of each *eEF1Bγ* homolog in mutant #4 by Sanger sequencing (Fig. 2d). *NbeEF1Bγ1* contained a 1-bp insertion, a 1-bp deletion, and a 1-bp substitution; in *NbeEF1Bγ2*, three of the clones sequenced had the wild-type (WT) sequence, with another two clones showing a 1-bp insertion of an A or G. Some of the clones sequenced for *NbeEF1Bγ3* also had the WT sequence, with another clone showing a 1-bp insertion of an A and another clone with a 1-bp substitution; in the clones sequenced for *NbeEF1Bγ4*, we obtained one clone with the WT sequence, with the remaining clones carrying a 1-bp insertion of a T or G. This analysis confirmed that mutant #4 is a chimera, harboring mutations in some or all copies of the four homologs. In addition, off-target effects were assessed in mutant #4, and no mutations were detected at the predicted off-target sites. Consequently, mutant #4, in which only the desired homologs were edited, was determined to be suitable for use in subsequent experiments.

### Dwarf phenotype and reduced mutant transcript levels of the *eEF1Bγ*-edited lines

**Fig. 3:**
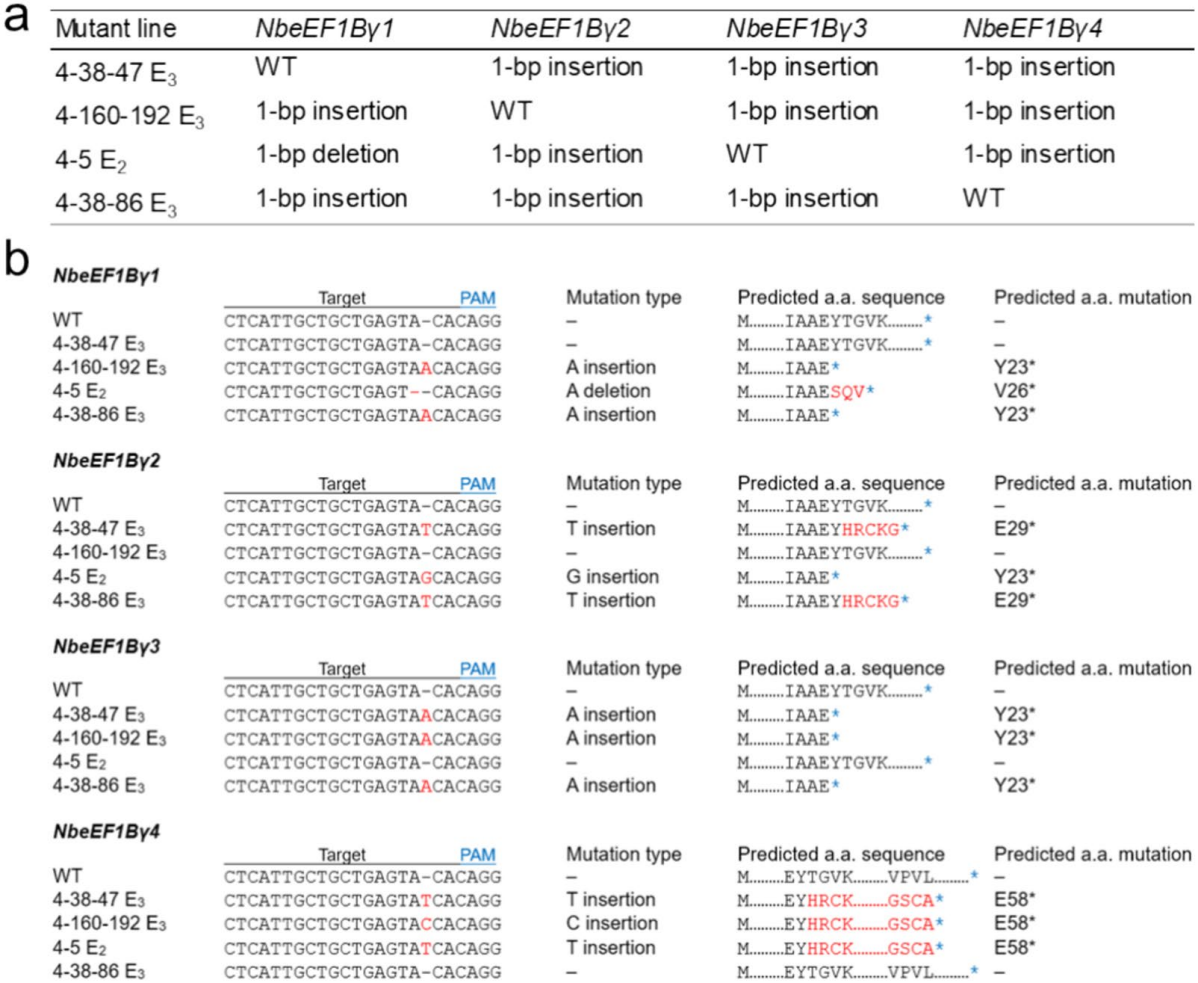
Mutation sequences and predicted amino acid sequences of the selected *eEF1Bγ*-edited lines in *N. benthamiana*. **a** Summary of the mutations in each of the *eEF1Bγ*-edited progeny from mutant #4, with mutations in all four *eEF1Bγ* homologs. Each *eEF1Bγ*-edited line contained one intact *eEF1Bγ* homolog with the remaining three harboring a 1-bp insertion or deletion. **b** DNA sequences (left) and predicted amino acid sequences and mutation (right) for each *eEF1Bγ* in the mutant lines. All 1-bp insertions or deletions in *eEF1Bγ* homologs induced premature stop codons. Black underlines indicate the sgRNA target site. Blue underlines indicate the PAM. Red font indicates the mutated sequences. * indicates a stop codon. *WT* wild-type plant

We allowed the plant derived from mutant #4 to produce seeds in order to obtain progeny plants with mutations in all *eEF1Bγ* homologs. We genotyped 222 E_1_ plants derived from mutant #4 for mutations in *eEF1Bγ* genes via restriction enzyme digestion; however, we failed to obtain quadruple mutants. We were also unsuccessful in obtaining a mutant line in which all homologs were mutated in later generations. For example, the plant *eEF1Bγ* 4–89 E_1_ contained a biallelic mutation in *NbeEF1Bγ1* and *NbeEF1Bγ4* and was heterozygous for a mutation in *NbeEF1Bγ2* and *NbeEF1Bγ3*. *NbeEF1Bγ2* and *NbeEF1Bγ3* are also located on chromosome 3, and one chromosome carries both mutant alleles (mutant *NbeEF1Bγ2* and mutant *NbeEF1Bγ3*), while the other chromosome carries both WT alleles (WT *NbeEF1Bγ2* and WT *NbeEF1Bγ3*). Among the 96 4–89 E_2_ progeny analyzed, only 8 plants carried heterozygous mutations in *NbeEF1Bγ2* and *NbeEF1Bγ3*, while the remaining 88 plants carried WT sequences for both homologs. No homozygous mutants for both *NbeEF1Bγ2* and *NbeEF1Bγ3* were identified. Therefore, we selected E_2_ and E_3_ mutant lines in which only one homolog retained the WT sequence, while the three remaining homologs contained homozygous mutations (Fig. 3a).

**Fig. 4:**
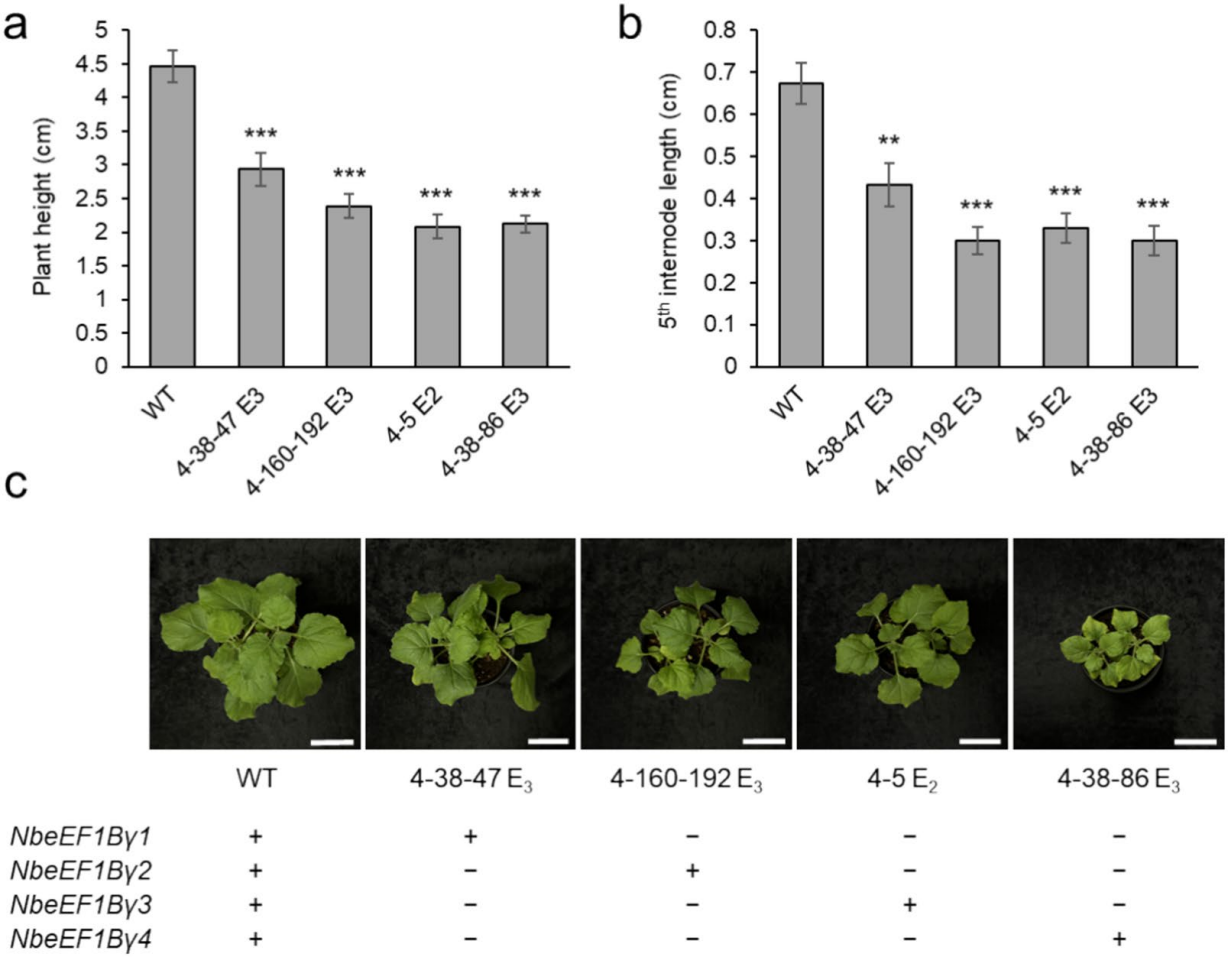
Dwarfism of the selected *eEF1Bγ*-edited lines. Plant height (**a**) and 5th internode length (**b**) were calculated. Data are presented as means ± standard error (SE, *n* = 21–24 for *eEF1Bγ*-edited plants; *n* = 22 for wild-type plants). Statistical significance was determined by a two-tailed *t* test in Microsoft Excel relative to WT: *P < 0.05, **P < 0.01, and ***P < 0.001. **c** Phenotypes of the *eEF1Bγ*-edited lines. Each pot contained two individual plants. “+ ” indicates WT sequence, and “–” indicates knockout mutation. *WT* wild-type plant. Scale bars, 5 cm

To determine the sequences of all *eEF1Bγ* genes in the selected mutant lines 4–38–47 E_3_, 4–160–192 E_3_, 4–5 E_2_, and 4–38–86 E_3_, we amplified the target region of each *eEF1Bγ* homolog and subjected them to Sanger sequencing. For each of the *eEF1Bγ*-edited lines, we confirmed that the mutant sequences were fixed in three of the four homologs (Fig. 3b). In 4–38–47 E_3_ plants, *NbeEF1Bγ1* was WT, whereas *NbeEF1Bγ2*, *NbeEF1Bγ3*, and *NbeEF1Bγ4* all contained a 1-bp insertion. In 4–160–192 E_3_ plants, *NbeEF1Bγ2* was the remaining functional copy, with a 1-bp insertion in *NbeEF1Bγ1*, *NbeEF1Bγ3*, and *NbeEF1Bγ4*. In 4–5 E_2_ plants, *NbeEF1Bγ3* was WT, while *NbeEF1Bγ1* had a 1-bp deletion, and *NbeEF1Bγ2* and *NbeEF1Bγ4* had 1-bp insertions. In 4–38–86 E_3_ plants, we detected the WT sequence in *NbeEF1Bγ4* and a 1-bp insertion in the other three homologs. The 1-bp insertions or deletions in each mutant line caused frameshifts, resulting in premature stop codons. The selected *eEF1Bγ*-edited lines showed dwarfism (Fig. 4). The plant height and 5th internode length of each mutant line were significantly smaller than the WT (Fig. 4a, b).

**Fig. 5:**
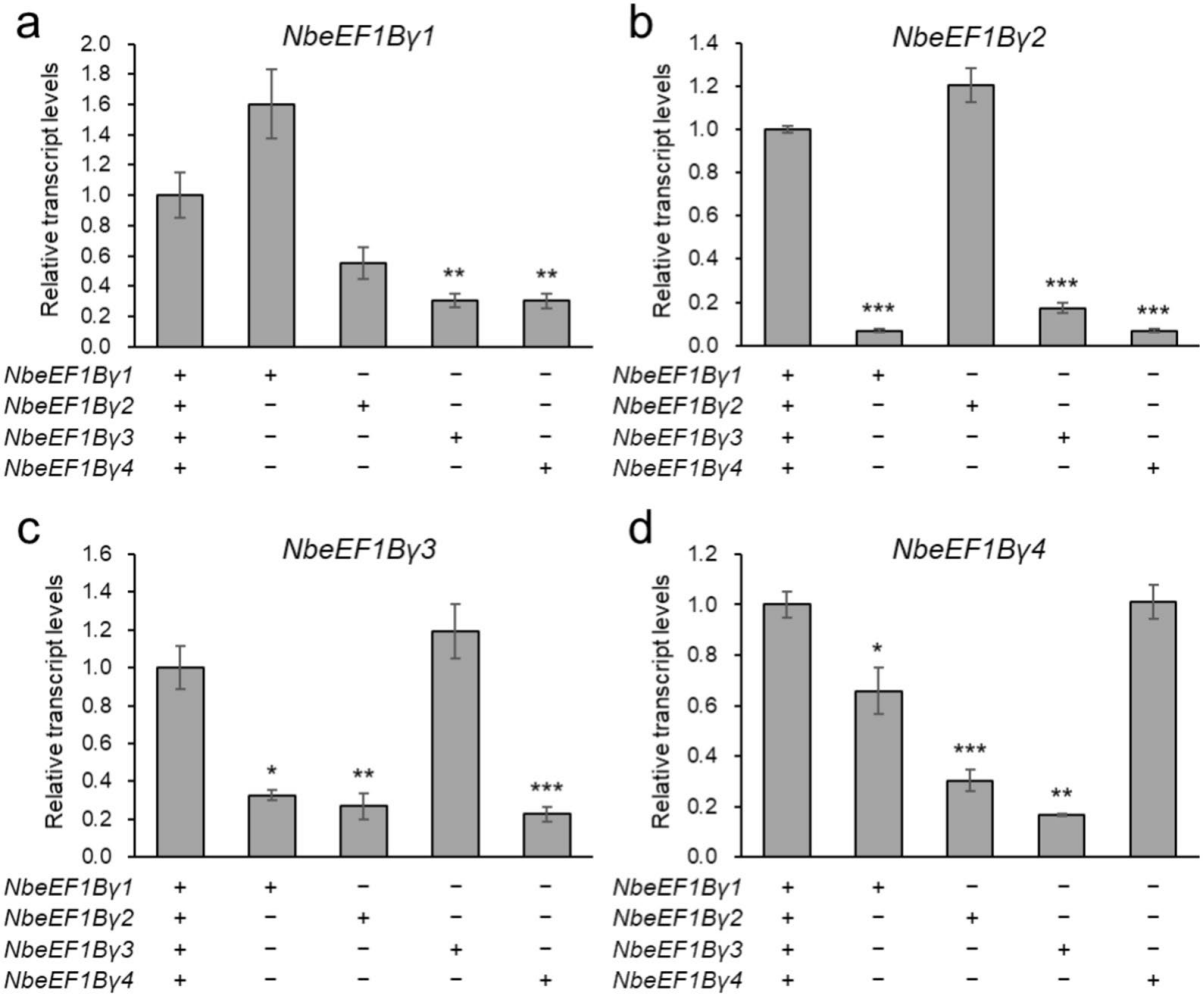
Reduction in expression levels of mutant *eEF1Bγ* homologs in the *eEF1Bγ*-edited lines. Relative transcript levels of Niben101Scf10540g00002.1 (**a**), Niben101Scf01552g05010.1 (**b**), Niben101Scf01552g05013.1 (**c**), and Niben101Scf03350g00002.1 (**d**) in wild-type plants and the 4–38–47 E_3_, 4–160–192 E_3_, 4–5 E_2_, and 4–38–86 E_3_ mutants. Data are presented as means ± SE (*n* = 4 for *eEF1Bγ*-edited plants; *n* = 3 for wild-type plants). Statistical significance was determined by a two-tailed *t* test in Microsoft Excel relative to WT: *P < 0.05, **P < 0.01, and ***P < 0.001

The above mutations in *eEF1Bγ* are expected to result in truncated proteins due to nonsense mutations. However, mutations in *eEF1Bγ* genes may lead to compensatory increases or decreases in expression of homologs with mutant sequences. To assess the consequences of genetically inactivating three of the four *eEF1Bγ* genes in *N. benthamiana*, we examined the expression levels of each homolog in the selected *eEF1Bγ*-edited lines, 4–38–47 E_3_, 4–160–192 E_3_, 4–5 E_2_, and 4–38–86 E_3_, by RT-qPCR (Fig. 5). In each *eEF1Bγ*-edited line, the remaining single functional homolog was expressed at a level similar to that of the WT, indicating that the one functional homolog did not compensate for the other knockout homologs. By contrast, the other three homologs with mutations showed significantly lower expression levels than the WT in almost all cases. This phenomenon may be attributed to the elimination of transcripts containing premature termination codons due to nonsense-mediated mRNA decay (NMD), one of several quality control mechanisms (Shaul 2015). Consequently, we obtained *eEF1Bγ*-edited lines with nonsense mutations in three of the four homologs and even reduced expression of these genes.

### Reduced TEV accumulation in the *eEF1Bγ*-edited plants

**Fig. 6:**
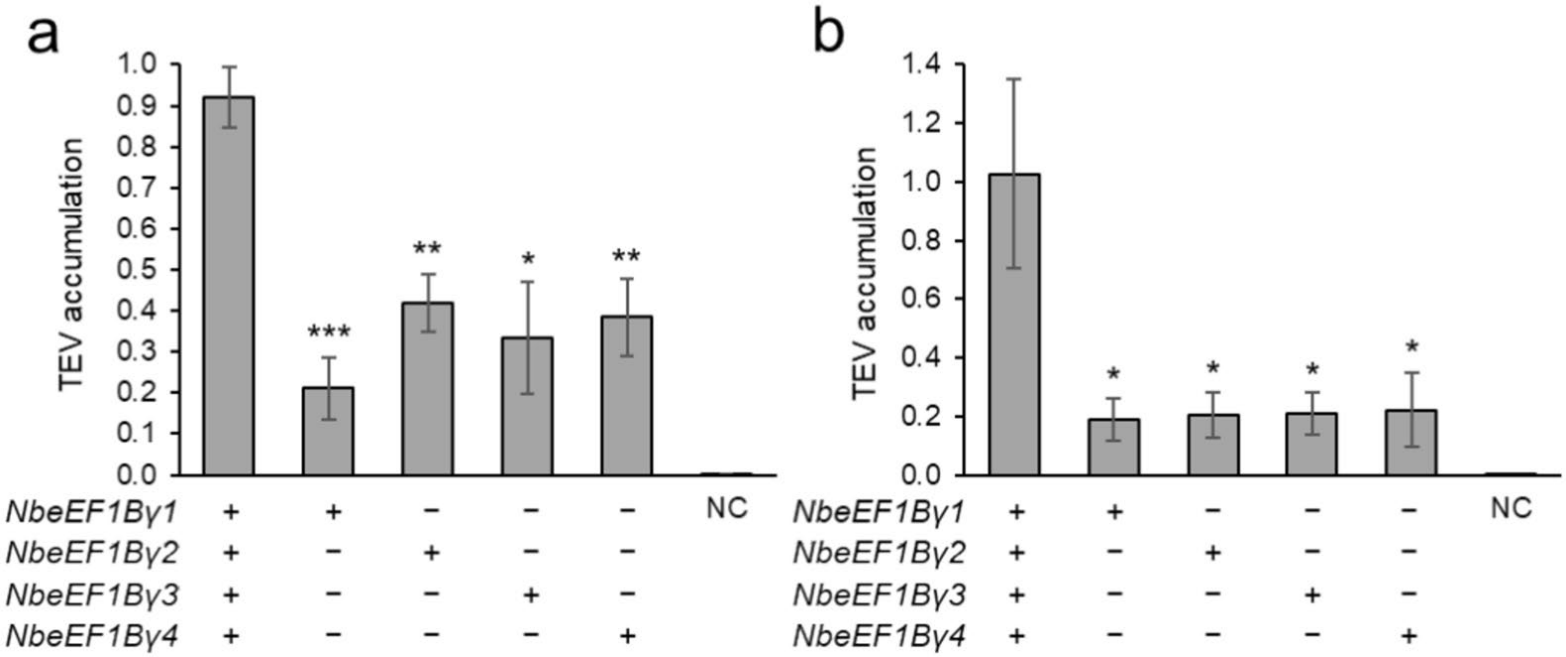
Reduced TEV accumulation in the *eEF1Bγ*-edited lines as determined by quantitative ELISA. Plants of the *eEF1Bγ*-edited lines, 4–38–47 E_3_, 4–160–192 E_3_, 4–5 E_2_, and 4–38–86 E_3_ were inoculated with TEV-HAT, and qELISA was conducted using inoculated leaves (**a**) and systemic leaves (**b**) at 5 DPI. Data are presented as means ± SE (*n* = 3–8). Statistical significance was determined by a two-tailed *t* test in Microsoft Excel relative to WT: *P < 0.05, **P < 0.01, and ***P < 0.001

To test whether these *eEF1Bγ*-edited lines exhibited virus resistance, we inoculated 4–38–47 E_3_, 4–160–192 E_3_, 4–5 E_2_, and 4–38–86 E_3_ lines with PVX, TMV, TBSV, or TEV. At 5 days post-inoculation (DPI), PVX disease symptoms, such as systemic leaf curling and necrosis, were observed in both WT and *eEF1Bγ*-edited plants (Fig. S5). To confirm PVX infection, we measured the accumulation of viruses in both inoculated and systemic leaves at 5 DPI by enzyme-linked immunosorbent assay (ELISA) (Fig. S6a, b). We detected similarly high PVX accumulation levels in inoculated leaves and systemic leaves from both *eEF1Bγ*-edited and WT plants. TMV-inoculated plants showed mild necrotic lesions and leaf curling, with no clear differences observed between WT and *eEF1Bγ*-edited plants (Fig. S5). There were no significant differences in TMV accumulation between *eEF1Bγ*-edited lines and the WT in either inoculated or systemic leaves, as determined by ELISA (Fig. S6c, d). Both *eEF1Bγ*-edited lines and the WT showed severe leaf curling in systemic leaves after TBSV inoculation, accompanied by uniform TBSV accumulation based on ELISA (Fig. S5, S6e, f). By contrast, *eEF1Bγ*-edited plants exhibited reduced TEV accumulation following TEV inoculation in both inoculated and systemic leaves compared to WT plants (Fig. S7). To accurately compare TEV accumulation, quantitative ELISA was conducted; *eEF1Bγ*-edited plants showed a 54.5–77.0% reduction in TEV coat protein (CP) accumulation in inoculated leaves compared to the WT (Fig. 6a). In addition, *eEF1Bγ*-edited plants showed a 78.4–81.7% reduction in TEV CP accumulation in systemic leaves (Fig. 6b). Despite these differences in TEV accumulation, there were no discernible phenotypic differences between WT and *eEF1Bγ*-edited plants after TEV inoculation (Fig. S5). Both WT and *eEF1Bγ*-edited plants showed severe necrotic lesions on inoculated leaves and mild leaf curling in systemic leaves after TEV inoculation.

## Discussion

Here, we used CRISPR/Cas9-mediated gene editing to target the *eEF1Bγ* genes in *N. benthamiana* to generate lines with virus resistance. We developed *eEF1Bγ*-edited lines using a TRV-mediated gene editing system (Ali et al. 2015), in which the sgRNA is delivered via a TRV vector into *Cas9*-overexpressing plants (Lee et al. 2024). We designed and constructed a pTRV2-sgRNA construct targeting all four *eEF1Bγ* genes of *N. benthamiana* (Fig. 1). tRNA-Ile has cell-to-cell mobility and facilitates efficient systemic editing in Arabidopsis and *N. benthamiana* when fused with sgRNA (Ellison et al. 2020; Nagalakshmi et al. 2022). Due to the mobility of tRNA-Ile, pTRV-*eEF1Bγ*-sgRNA-inoculated plants had a gene editing efficiency of 14.3–24.6% in systemic leaves (Fig. 2a). We wanted to obtain a homozygous quadruple mutant from mutant #4 with mutations in all *eEF1Bγ* homologs through generation advancement (Fig. 2c, d). Arabidopsis contains two *eEF1Bγ* homologs. Among Arabidopsis T-DNA insertion mutants, no homozygous double mutants with insertions in the exon regions of the two *eEF1Bγ* genes could be produced (Lohmann et al. 2023). Similarly, in *N. benthamiana*, we failed to identify plants with simultaneous knockout of all four *eEF1Bγ* homologs. Therefore, we selected edited lines with mutations in all but one *eEF1Bγ* in all possible combinations (Fig. 3a). All the mutations detected in the selected mutant lines resulted in premature stop codons, leading to the predicted loss of *eEF1Bγ* function (Fig. 3b).

Since the copy number of *eEF1Bγ* genes in *N. benthamiana* was not previously known, we identified *eEF1Bγ* homologs in *N. benthamiana*. Based on the amino acid sequences of the proteins encoded by two *eEF1Bγ* genes (At1g09640 and At1g57720) in Arabidopsis, we obtained four transcripts (Niben101Scf10540g00002.1 [*NbeEF1Bγ1*], Niben101Scf01552g05010.1 [*NbeEF1Bγ2*], Niben101Scf01552g05013.1 [*NbeEF1Bγ3*], and Niben101Scf03350g00002.1 [*NbeEF1Bγ4*]) predicted to encode proteins similar to the Arabidopsis EF1Bγ proteins used as query sequences. We confirmed their expression by exploiting public transcriptome data (Fig. S4). *NbeEF1Bγ2* and *NbeEF1Bγ3* are located on chromosome 3, while *NbeEF1Bγ1* and *NbeEF1Bγ4* are located on chromosome 10. A phylogenetic tree based on eEF1Bγ protein sequences in the four species, *N. benthamiana*, tomato, pepper, and Arabidopsis, suggests a closer relationship between *N. benthamiana* and tomato and pepper compared to Arabidopsis (Fig. S1). A comparison of eEF1Bγ protein sequences in the four plant species revealed a high level of similarity (greater than 76%), particularly within the Solanaceae family, where the similarity was greater than 86% (Table S2). These findings indicate that eEF1Bγ proteins are highly conserved across these species.

We generated *eEF1Bγ*-edited lines with 1-bp insertions or deletions in the 2nd exons of most of the four *N. benthamiana eEF1Bγ* homologs (Fig. 3). Despite the presence of biallelic mutations in each *eEF1Bγ* homolog in mutant #4 (Fig. 2c, d), we obtained no progeny with homozygous quadruple mutations, even in the E_2_ and E_3_ generations. Furthermore, homozygous quadruple mutants were not observed from the *eEF1Bγ* 4–89 E_1_ mutant, which contained homozygous mutations in *NbeEF1Bγ1* and *NbeEF1Bγ4* and heterozygous mutations in *NbeEF1Bγ2* and *NbeEF1Bγ3*. The skewed segregation ratio (0:1:11.5) suggests that the *eEF1Bγ* quadruple mutant may be lethal and that plants carrying mutant alleles for both *NbeEF1Bγ2* and *NbeEF1Bγ3* exhibit developmental defects. As an alternative, we selected lines for three out of the four homologs. These lines displayed reduced plant growth compared to WT plants (Fig. 4). In Arabidopsis, the two *eEF1Bγ* homologs act redundantly and are involved in plant development (Lohmann et al. 2023). The results of this study suggest that all four conserved *eEF1Bγ* homologs may function redundantly in *N. benthamiana*. Moreover, the inability to obtain homozygous quadruple *eEF1Bγ*-edited lines and the observation that the selected *eEF1Bγ*-edited lines showed dwarfism suggest an essential role of eEF1Bγ in *N. benthamiana* development.

Studies in other plant species have implicated eEF1Bγ in the replication of RNA viruses such as TMV, TBSV, and PVX (Sasvari et al. 2011; Hwang et al. 2013, 2015). In the current study, unlike VIGS-mediated knockdown studies, we knocked out three of the four *eEF1Bγ* homologs in *N. benthamiana* via VIGE and tested the resistance of these plants to the viruses TMV, PVX, TBSV, and TEV. The selected *eEF1Bγ*-edited lines did not show resistance to TMV, PVX, or TBSV (Fig. S5, S6). This suggests that a single functional *eEF1Bγ* homolog was sufficient to function in the replication of these viruses due to the redundancy of eEF1Bγ, or that genetic compensation mechanisms may have compensated for the knockout of *eEF1Bγ*, resulting in no observable differences in virus replication. Compensatory networks are known to be activated to buffer against deleterious mutations, while such genetic compensation does not occur during translational or transcriptional knockdown (Rossi et al. 2015). In addition, when mutations cause degradation of the corresponding mRNA, related genes are triggered to undergo genetic compensation (El-Brolosy et al. 2019). In this context, the *eEF1Bγ* mutation could be considered deleterious due to its association with developmental processes (Fig. 4), and we also observed significantly reduced expression of the mRNA from the mutated homolog (Fig. 5). Therefore, genetic compensation that did not occur during VIGS-mediated knockdown of *eEF1Bγ* may have been activated in response to the mutation, leading to no significant impact on the replication of viruses such as TMV, TBSV, and PVX. In contrast to these viruses, the *eEF1Bγ*-edited lines showed partial resistance to TEV, with less virus accumulation compared to WT in both inoculated and systemic leaves (Fig. 6 and Fig. S6). This indicates a virus-specific dependency on eEF1Bγ for replication, highlighting its specific role in the replication of TEV in *N. benthamiana*. The correlation between eEF1Bγ and the TEV spread suggests that eEF1Bγ is a host factor responsible for TEV resistance conferred by CRISPR/Cas9-mediated editing.

Extending the finding that plant RNA viruses require host factors to complete their lifecycles (Wang 2015), our study contributes to ongoing efforts to develop virus-resistant crops through gene editing. Our findings suggest that precise gene editing of *eEF1Bγ* could offer a strategy for achieving durable recessive resistance, similar to approaches utilized for resistance to potyviruses (Bastet et al. 2019). To develop virus-resistant crops through gene editing of host factors, genome-wide CRISPR/Cas systems can be employed to identify host susceptibility and resistance factors (Mahas and Mahfouz 2018). This deepens our understanding of virus biology and enables the engineering of plant immunity. However, the knockout of a host factor may impair plant survival or cause developmental defects, underscoring the need to balance virus resistance with host viability and highlighting potential challenges and opportunities in deploying gene editing for crop protection. Instead of completely disrupting host factors critical for both viral replication and plant survival, introducing selective point mutations in *eEF1Bγ* to disrupt its interaction with TEV could provide resistance without affecting the survival or development of the host plant. This approach has been successfully demonstrated in Arabidopsis, where base editing of the pathogen susceptibility factor eIF4E mimicked a natural polymorphism to confer resistance to potyviruses (Bastet et al. 2019). Similarly, identifying the critical regions in the interaction between eEF1Bγ and TEV could enable precise gene editing strategies, offering durable and functional resistance while maintaining plant viability.

## Supplementary Information

Below is the link to the electronic supplementary material.

Supplementary file1 (DOCX 2773 KB)

## Data Availability

The data supporting the findings of this study are available from the corresponding author upon reasonable request.
