# Supplementary material for: CRISPR/Cas9-mediated editing of eukaryotic elongation factor 1B gamma (eEF1Bγ) reduces Tobacco etch virus accumulation in Nicotiana benthamiana
Source: Plant Cell Rep. 2025 Feb 22;44(3):62. doi: 10.1007/s00299-025-03440-x (PMC11846736; doi:10.1007/s00299-025-03440-x)
Supplement: Supplementary file 1 — Supplementary file1 (DOCX 2773 KB) [file 299_2025_3440_MOESM1_ESM.docx]

**Supplementary Figures**


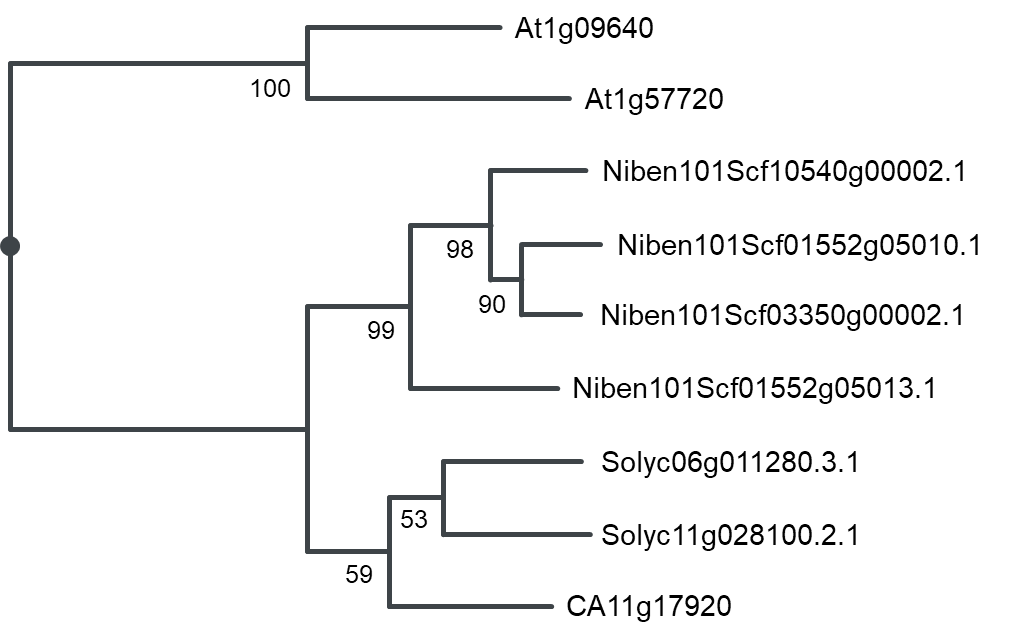


**Fig. S1** Phylogenetic tree of eEF1Bγ proteins from four different plant species. The phylogenetic tree was constructed with the neighbor-joining method using PID in the Jalview program.


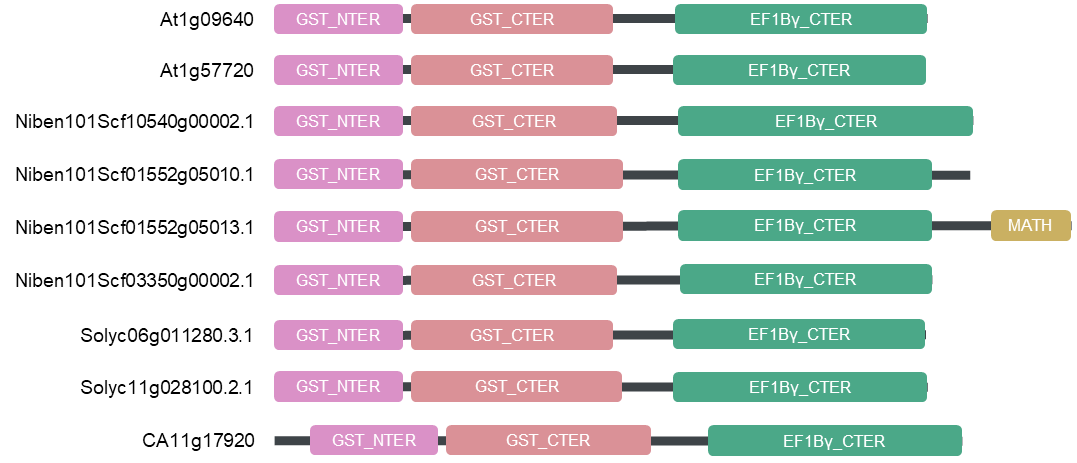


**Fig. S2** Diagram of the eEF1Bγ domain structure. The domain structure of eEF1Bγ proteins was predicted by the InterPro program. GST_NTER, glutathione S-transferase N-terminal domain; GST_CTER, glutathione S-transferase C-terminal domain; EF1Bγ_CTER, Elongation factor 1B gamma C-terminal domain; MATH, meprin and TRAF homology domain.


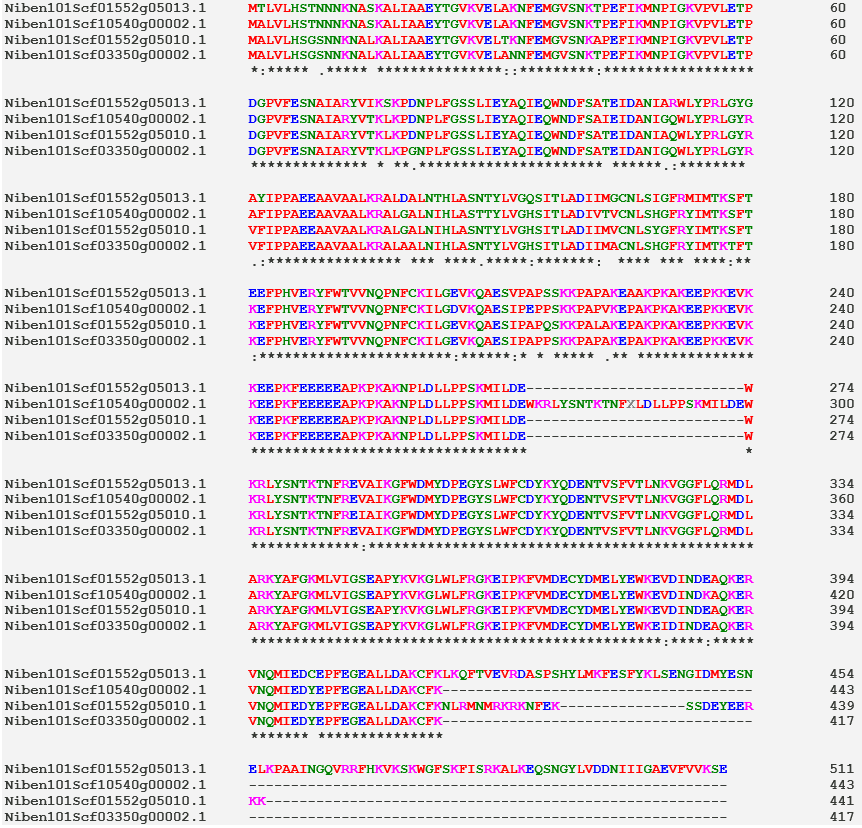


**Fig. S3** Alignment of amino acid sequences of the eEF1Bγ proteins in *N. benthamiana*. The protein sequences of all four *N. benthamiana* eEF1Bγs were aligned using the Clustal Omega program.


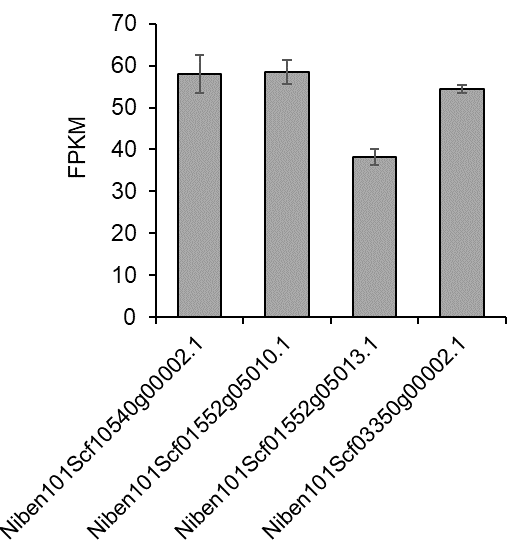


**Fig. S4**. Expression analysis of *eEF1Bγ* homologs in *N. benthamiana*. Transcriptome data from three SRA files (SRR11747765, SRR11747766, and SRR11747767) corresponding to *N. benthamiana* leaf samples were used. Fragments Per Kilobase per Million mapped fragments (FPKM) values were calculated for each of the four *eEF1Bγ* homologs. Data are presented as means ± SD. *n* = 3.


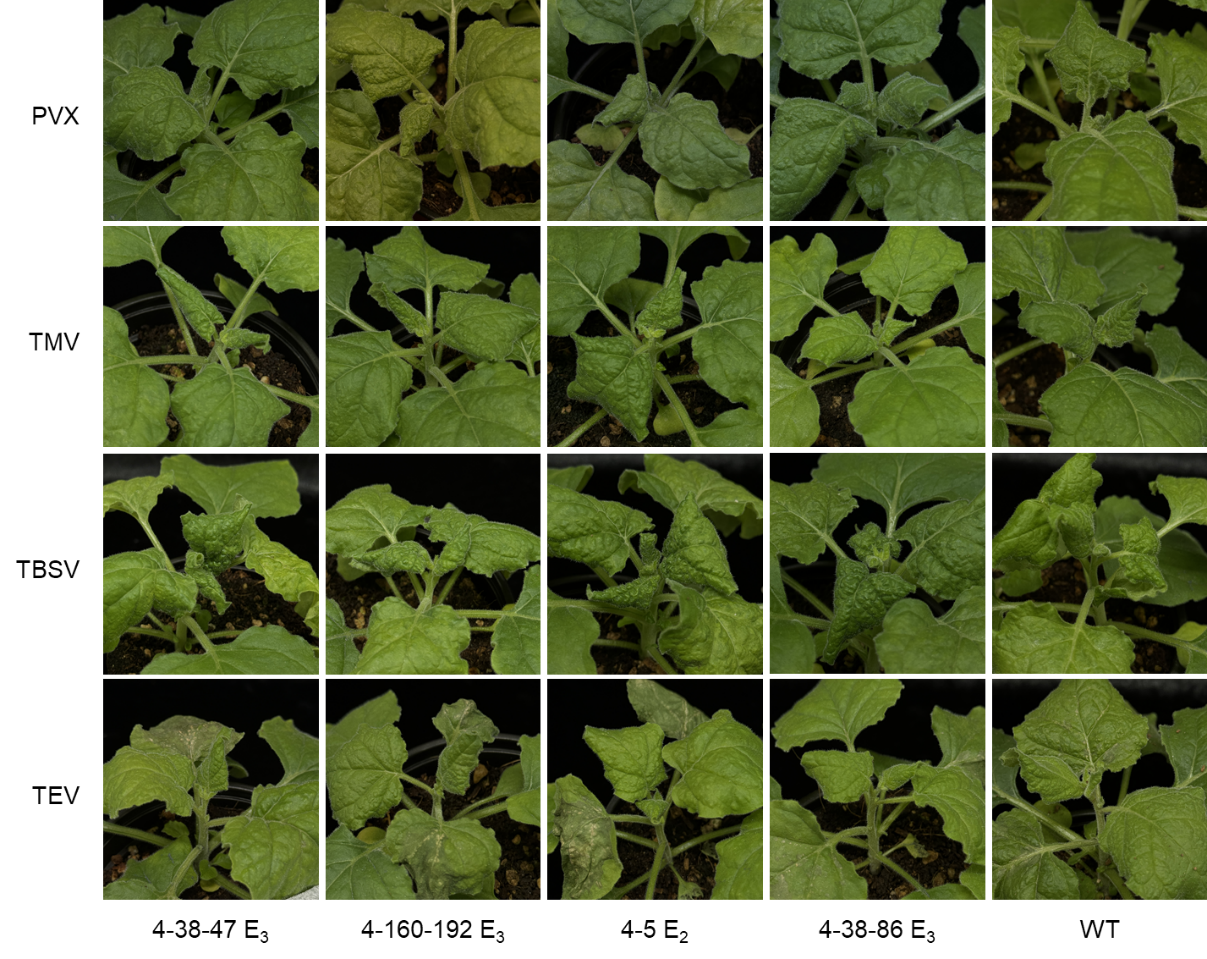


**Fig. S5** Virus symptoms of *eEF1Bγ*-edited lines and WT plants inoculated with PVX, TMV, TBSV, and TEV. The photographs display virus symptoms observed at 5 DPI.


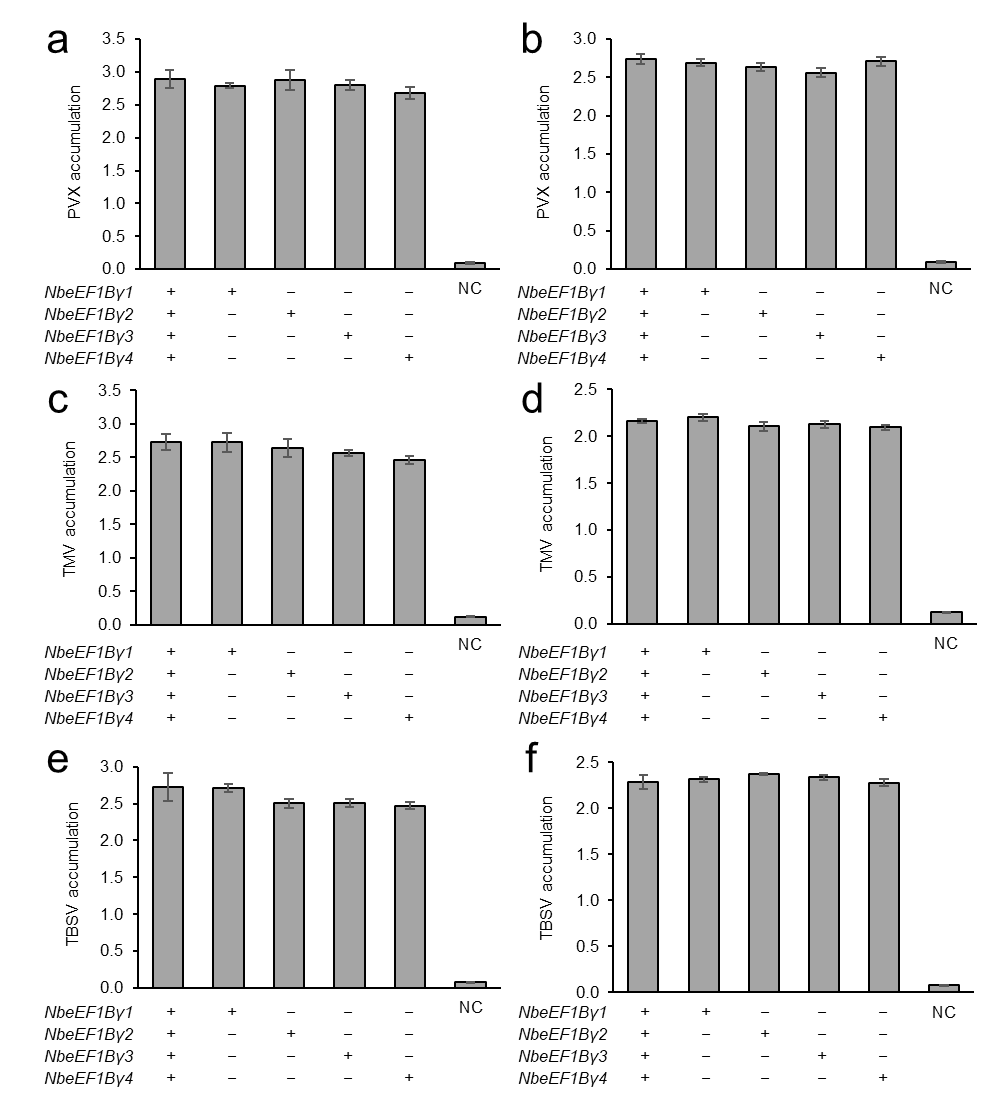


**Fig. S6** Resistance analysis of *eEF1Bγ*-edited lines inoculated with PVX, TMV, or TBSV. Plants of the *eEF1Bγ*-edited lines 4-38-47 E_3_, 4-160-192 E_3_, 4-5 E_2_, and 4-38-86 E_3_ were inoculated with PVX (**a and b**), TMV (**c and d**), or TBSV (**e and f**), and ELISA was conducted using inoculated leaves (**a, c, and e**) and systemic leaves (**b, d, and f**) at 5 DPI. Data are presented as means ± SE (*n* = 4–8).


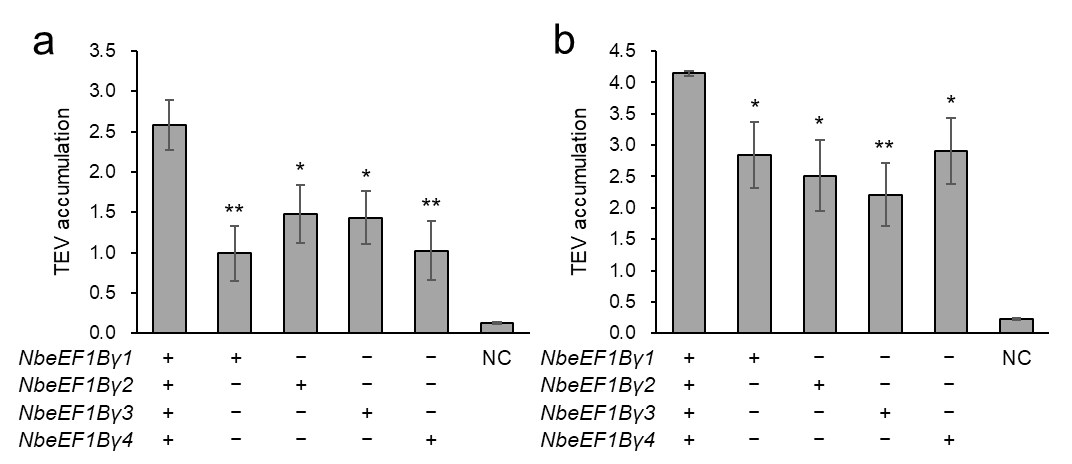


**Fig. S7** Resistance analysis of *eEF1Bγ*-edited lines inoculated with TEV. Plants of the *eEF1Bγ*-edited lines, 4-38-47 E_3_, 4-160-192 E_3_, 4-5 E_2_, and 4-38-86 E_3_ were inoculated with TEV-HAT, and ELISA was conducted using inoculated leaves (**a**) and systemic leaves (**b**) at 5 DPI. Data are presented as means ± SE (*n* = 10–12 for *eEF1Bγ*-edited plants or wild-type plants; *n* = 4 for non-inoculated plants, negative control, NC). Statistical significance was determined by a two-tailed t-test in Microsoft Excel relative to WT: *, *P* < 0.05, **, *P* < 0.01, and ***, *P* < 0.001.

**Supplementary Tables**

**Table S1** List of primers used in this study.

| Primer name | Sequence (5' to 3') | Amplicon size (bp) | Purpose |
| --- | --- | --- | --- |
| eEF1Bγ-sgRNA-MfeI-F | GATACAATTGCTCATTGCTGCTGAGTACACGTTTTAGAGCTAGAAATA | 218 | pTRV2-sgRNA vector construction |
| tRNAIle-XmaI-R | GTACCCCGGGCTGCAGCCATGGAAAAAATGCTTCCGGCGGGGCTCG |  |  |
| eEF1Bγ1-PCR-RE-F | GATGAAATTTCTTGATGATATCCTCGC | 604 | Mutation detection |
| eEF1Bγ1-PCR-RE-R | ATTTGAGCCTGTCAAGGAAAAAGA |  |  |
| eEF1Bγ2-PCR-RE-F | TGGGACATTTTGCATGAAGGCT | 650 |  |
| eEF1Bγ2-PCR-RE-R | CCCGCCTGCCATTCCTACTA |  |  |
| eEF1Bγ3-PCR-RE-F | GGGATAAAATTTCTTGAAGATATCCTCGT | 468 |  |
| eEF1Bγ3-PCR-RE-R | AAAAGGGGATTGTCGGGCTT |  |  |
| eEF1Bγ4-PCR-RE-F | TTTCTCTTGGGTAGGTAATTTCTTGATG | 478 |  |
| eEF1Bγ4-PCR-RE-R | GGATTGCCGGGCTTCAATTT |  |  |
| eEF1Bγ1 qRT-F | CAAGTTTCAACTGTGCAACCATGG | 158 | RT-qPCR |
| eEF1Bγ1 qRT-R | GATAAACTCAGGTGTCTTGTTTGATACC |  |  |
| eEF1Bγ2 qRT-F | GGCACCTGAGTTTATCAAGATGAAC | 122 |  |
| eEF1Bγ2 qRT-R | GATTGTCGGGCTTCAATTTAGTTACATAT |  |  |
| eEF1Bγ3 qRT-F | ACAAGACACCTGAGTTTATCAAGATGAAT | 167 |  |
| eEF1Bγ3 qRT-R | CTCGATTTGAGCATATTCAATCAATGATGAA |  |  |
| eEF1Bγ4 qRT-F | GCACGCTATGTAACTAAATTGAAGCCCGG | 138 |  |
| eEF1Bγ4 qRT-R | ACGTGGGTACAACCATTGCC |  |  |
| Nb-actin-F | CTGAGAGATTCCGCTGC | 143 |  |
| Nb-actin-R | GAGGACAATGTTTCCGTAC |  |  |

**Table S2** Similarity among eEF1Bγ proteins from four plant species.

| Similarity (%) | At1g09640 | At1g57720 | Solyc06g011280.3.1 | Solyc11g028100.2.1 | CA11g17920 | Niben101Scf10540g00002.1 | Niben101Scf01552g05010.1 | Niben101Scf01552g05013.1 | Niben101Scf03350g00002.1 |
| --- | --- | --- | --- | --- | --- | --- | --- | --- | --- |
| At1g09640 | – | 93 | 85 | 85 | 85 | 80 | 85 | 82 | 82 |
| At1g57720 | – | – | 85 | 84 | 83 | 76 | 82 | 82 | 81 |
| Solyc06g011280.3.1 | – | – | – | 96 | 93 | 86 | 93 | 92 | 91 |
| Solyc11g028100.2.1 | – | – | – | – | 93 | 88 | 94 | 92 | 92 |
| CA11g17920 | – | – | – | – | – | 82 | 91 | 91 | 89 |
| Niben101Scf10540g00002.1 | – | – | – | – | – | – | 91 | 90 | 91 |
| Niben101Scf01552g05010.1 | – | – | – | – | – | – | – | 95 | 98 |
| Niben101Scf01552g05013.1 | – | – | – | – | – | – | – | – | 95 |
| Niben101Scf03350g00002.1 | – | – | – | – | – | – | – | – | – |

**Table S3** List of primers used in off-target analysis.

| Predicted off-target sequence | Primer name | Sequence (5' to 3') | Amplicon size (bp) |
| --- | --- | --- | --- |
| CTCAGTACTGCTGAAAACACAGG | eEF1Bγ-off1-F | GTGGTCCACGACTCTAAGCA | 502 |
|  | eEF1Bγ-off1-R | CCTGATTGCTCATCAGAGAATACTAAATCAG |  |
| CCTATTGCTCCTAAGTACACAGG | eEF1Bγ-off2-F | CATTGTACCTTGTTGCCCAGCTA | 664 |
|  | eEF1Bγ-off2-R | TTCGGTGAAACGCTTACCCT |  |
| AGCATTGCTGCTGACAACACTGG | eEF1Bγ-off3-F | AAAGAGTAGGAGATCTGCAGCC | 599 |
|  | eEF1Bγ-off3-R | ACGGGAGAGGATCTACCAGTT |  |
| TTCATTGCTGCTCAGAAAACAGG | eEF1Bγ-off4-F | TACCGCTGATAGGACAGAAGC | 562 |
|  | eEF1Bγ-off4-R | GATAGTTGTTGTTGTTATTGTTGTTATGCCCA |  |
| CTCATTGCCGCAGAGGACACAGG | eEF1Bγ-off5-F | GGAGTATTAGCCCCGCATG | 551 |
|  | eEF1Bγ-off5-R | ATCATACATCTTTGAAAGGTCGCTGAG |  |
| CTCATAGATGGTGAGTACACGGG | eEF1Bγ-off6-F | CTTCATGTCCTAGGCGGCA | 731 |
|  | eEF1Bγ-off6-R | GGAGCATCTATAGTTGGAGCCTGATA |  |
| CTCATTGATGCTGAGTACACGAG | eEF1Bγ-off7-F | TACAAACTGTCAATATCTAGGCCAATGTCTAAT | 789 |
|  | eEF1Bγ-off7-R | GGAGTCATCGAGGTAGCTGTAA |  |
